# Drug selection for inner ear therapy

**DOI:** 10.3389/fphar.2024.1452927

**Published:** 2024-10-17

**Authors:** Alec N. Salt, Jeremy G. Turner

**Affiliations:** Turner Scientific, Jacksonville, IL, United States

**Keywords:** molecule, lipophilicity, polarity, perilymph, elimination, distribution

## Abstract

**Introduction:**

One of the primary tenets in pharmacotherapy is that the applied drug must reach the target tissue at therapeutic concentration. For many therapies intended to treat hearing disorders it has become apparent that we have failed to achieve this goal, contributing to poor outcomes in several important clinical trials. The crux of the delivery problem is that small lipophilic molecules pass with relative ease through membranous boundaries of the body. This initially seems advantageous when the drug is applied intratympanically, enabling entry into perilymph through the round window membrane. Unfortunately, the same property also allows the drug to pass through endothelial cells of blood capillaries, allowing it to be eliminated from perilymph. Drugs that are eliminated rapidly as they diffuse along the cochlear scalae will only treat basal high-frequency cochlear regions and will not reach therapeutic concentrations in the apical regions of the human cochlea.

**Methods:**

We have used the FluidSim program, a computer model of the inner ear fluids, to derive perilymph elimination properties for 15 molecules from published and archival data sets, which are compared with calculated molecular properties.

**Results:**

Smaller, lipophilic drugs are shown to be eliminated from perilymph more rapidly, with half-times as fast as 17 min, compared to larger, polar ones, with half-times as long as 1,304 min (21.7 h).

**Discussion:**

Based on their molecular properties’ drugs can be identified that distribute well along the cochlea when applied intratympanically. This excludes many drugs that have been used for, or are currently in development for, inner ear therapy. On the other hand, it opens a vast array of less-studied, larger molecules, many of which would be unsuitable for oral delivery (characterized as “not druglike”) but representing promising candidates for local inner ear therapy. In the earliest stages of consideration, drugs need to be selected based on the properties which govern their ability to reach the appropriate target site and not whether they are efficacious in small animals or have high potency *in vitro*. Confirmation that the selected drug is reaching the target site(s) in a large animal model should ideally precede expensive clinical trials.

## 1 Introduction

Over the last decade we have developed a better understanding of the pharmacokinetic (PK) properties of small drug molecules in the ear. In the light of this knowledge, it has become apparent that the drugs currently in use for common clinical therapies exhibit PK properties not well suited for their intended purpose. For dexamethasone, the rate of elimination from perilymph is high with a measured half-time from scala tympani (ST) of guinea pigs in the range of 23–52 min (Salt et al., 2012; Salt et al., 2018; present study). This rate of loss during the diffusion of drug along the scalae restricts apical distribution along the cochlear spiral. Comparable elimination from the longer, human cochlea would result in only low concentrations reaching the apical half of the cochlea responsible for coding frequency regions important for hearing speech. In contrast, studies of the PK properties of gentamicin showed that gentamicin was better retained in perilymph with a measured ST elimination half time of 180–220 min (Salt et al., 2016; present study). The slower elimination allows gentamicin to distribute further apically along the cochlea. Unfortunately, this is an undesirable property when preservation of hearing is a major concern. Both drug therapies were established before their PK properties were measured and the molecules were selected no consideration of their likely distribution characteristics in the human ear.

In the larger field of systemic pharmacology, there have been major advances in the understanding of the relationship between the physical properties of drugs and their ability to reach target tissues. There was greatest interest in selecting drugs for their ability to reach tissues when delivered orally, commonly known as oral bioavailability. A widely used guideline followed by the pharmaceutical industry was the “Lipinski Rule of 5” (Lipinski, 2000), also known as the Pfizer Rule of Five. It was based on the observation that most drugs that are effective with oral delivery have physicochemical properties that fall within a specific area, specifically.

1) They have a molecular weight less than 500 Daltons.
2) Their lipophilicity (LogP) measures less than 5.
3) They have no more than 5 hydrogen bond donors (electronegative groups such as R-OH) and no more than 10 hydrogen bond acceptors (highly electronegative groups such as R-C=O).

**FIGURE 1:**
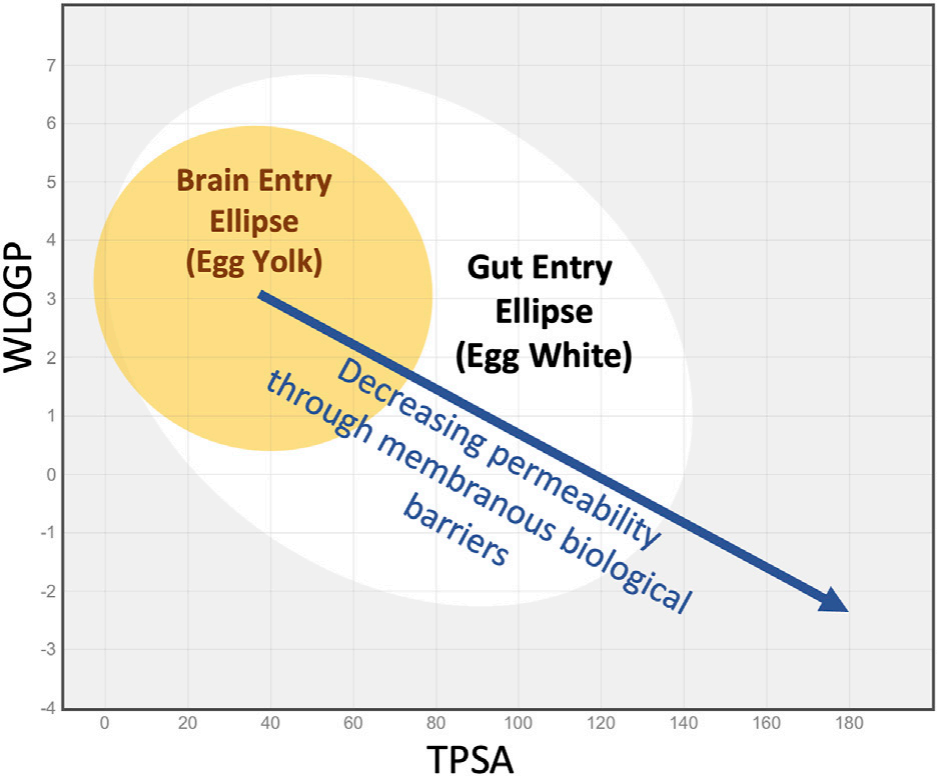
The “egg plot” adapted from Daina and Zoete, 2016, as generated by the SwissADME.com website (Daina et al., 2017) and based on Egan’s egg (Egan et al., 2000). The colored ellipses represent statistical boundaries based on intestinal absorption data for 660 small molecules (white) and for blood/brain entry of 260 small molecules (yellow) in humans. In general, small lipophilic molecules (low TPSA, high WlogP, upper left quadrant) can enter through the gut epithelium and also pass through the tighter blood-brain barrier. More polar, less lipophilic molecules (middle of plot, in the white ellipse) may enter through the gut epithelium but not the brain. Highly polar molecules that lie outside the white ellipse (right side of plot and extending beyond plotted range) may not enter the systemic circulation when given orally. The boundaries shown are statistical and exceptions commonly occur. Nevertheless, as lipophilicity decreases and TPSA increases, molecules tend to pass less easily through the lipid membranes of barrier epithelia.

Hydrogen bonding is a crucial intermolecular force influencing the 3D structure of molecules and their interaction with water. Compounds that violate Lipinski’s rules were likely to have poor absorption or permeation, leading to lower oral bioavailability, and poor aqueous solubility. Solubility is also a major consideration because the drugs need to dissolve to be absorbed. Molecular characterization was later refined by including the topological polar surface area (TPSA), a calculated measure of the area of the molecule covered in polar groups (charged groups, including hydrogen bond acceptors) (Kenny, 2022). A plot of lipophilicity (WlogP) against TPSA has become known as an “egg plot”, as illustrated in Figure 1 (Egan et al., 2000; Daina and Zoete, 2016). The ellipses are statistical boundaries based on the entry characteristics of hundreds of molecules in human studies. Molecules with properties within the “egg white” generally pass through the gut epithelium, from the gut lumen to the vasculature. Molecules falling within the “egg yolk” can pass through both the gut epithelium and the tighter blood-brain barrier, allowing them to enter the brain from the vasculature. Some molecules behave as exceptions to this general principle.

The relevance of the egg plot to inner ear PK is based on the following principles.

1) Ease of passage of molecules across membranous barriers of the body depends on the physical properties of the molecule.
2) Different barriers have different permeability properties, such as the gut, the blood-brain barrier, and the blood-labyrinth barrier. The membranous barriers relevant to the ear include the round window membrane, the mucosa covering the oval window at the stapes, the endothelial cells of capillaries (making up the blood-labyrinth barrier), and the endolymph-perilymph barrier (reviewed in Salt and Hirose, 2018).
3) Elimination from perilymph of the ear will have different characteristics compared to entry at the gut or blood-brain barrier. Entry and elimination processes have different characteristics and are not equivalent.
4) The egg plot published by Daina and Zoete, (2016) is based on binary data (whether the drug enters or does not enter at the specified boundary). In this study we have correlated molecular properties with a continuous variable, specifically the elimination half-time, as a measure of how rapidly the drug was lost from perilymph.

The implications of egg plot parameters to drug distribution in the inner ear have been discussed in prior review articles (Salt and Plontke, 2018; Salt and Plontke, 2020; Gay et al., 2022) and in more recent publications of the experimental studies on which this analysis is based, detailed below.

In the current study we used FluidSim software (v 5.0) to reanalyze archival data collected in the prior 12 years over which elimination rates of 15 drugs were measured using identical methodology. In that time there have been substantial changes to the FluidSim program and changes in the procedures used to derive elimination rates from the data. This reanalysis allowed us to use a standardized fitting procedure across all molecules, making all the elimination measurements directly comparable.

## 2 Methods

The core routines of the FluidSim program were developed in the mid-1980s to interpret experimental flow-marker measurements in the inner ear fluids. Marker concentrations were followed in time using ion-selective microelectrodes sealed into the cochlear fluid spaces. Simulations clearly demonstrated that marker distribution with time was dominated by passive diffusion, superimposed upon which were losses to other compartments, including the vasculature, and volume flow at very low rates. Over the years, FluidSim was trained on numerous marker studies, establishing flow rates, communication properties with adjacent fluid and tissue compartments, and elimination rates (loss of markers from perilymph to the vasculature). In the early 2000s, analysis with FluidSim became a valuable tool to interpret PK measurements when drugs were applied locally to the ear. The algorithms and knowledge incorporated into FluidSim are currently based on at least 40 published studies where FluidSim output was compared with measured data from inner ear fluids (Presented in Supplementary Table S1), and from a variety of other projects, presentations and proprietary contract studies. FluidSim calculations are based on the underlying physical processes by which drugs enter the ear and distribute through fluid and tissue compartments. The calculations simply move solute either within the compartment or between compartments depending on the prevailing concentration gradients driving the movement. FluidSim provides a perspective of what is physically possible, or impossible, in terms of drug distribution in the ear.

Calculations have shown that drug distribution in the ear depends strongly on the physical dimensions, whether it is a mouse, guinea pig or human. Quantitative dimensions of 8 species, including humans, are incorporated into FluidSim. Each fluid or tissue compartment is represented as an array of cross-sectional area values, with each value corresponding to a segment 0.1 mm in length. The mouse ST is relatively short, just 4.3 mm long (represented as 43 elements) while the human spiral ligament, which follows a long, wide spiral, is 47.3 mm long (represented as 473 elements). For each species, the cochlea compartments include ST, the endolymphatic space, scala vestibuli (SV) (including the vestibule), spiral ganglion, spiral ligament and the organ of Corti. Vestibular structures are also defined and calculated but have little influence over drug distribution along the cochlea.

**FIGURE 2:**
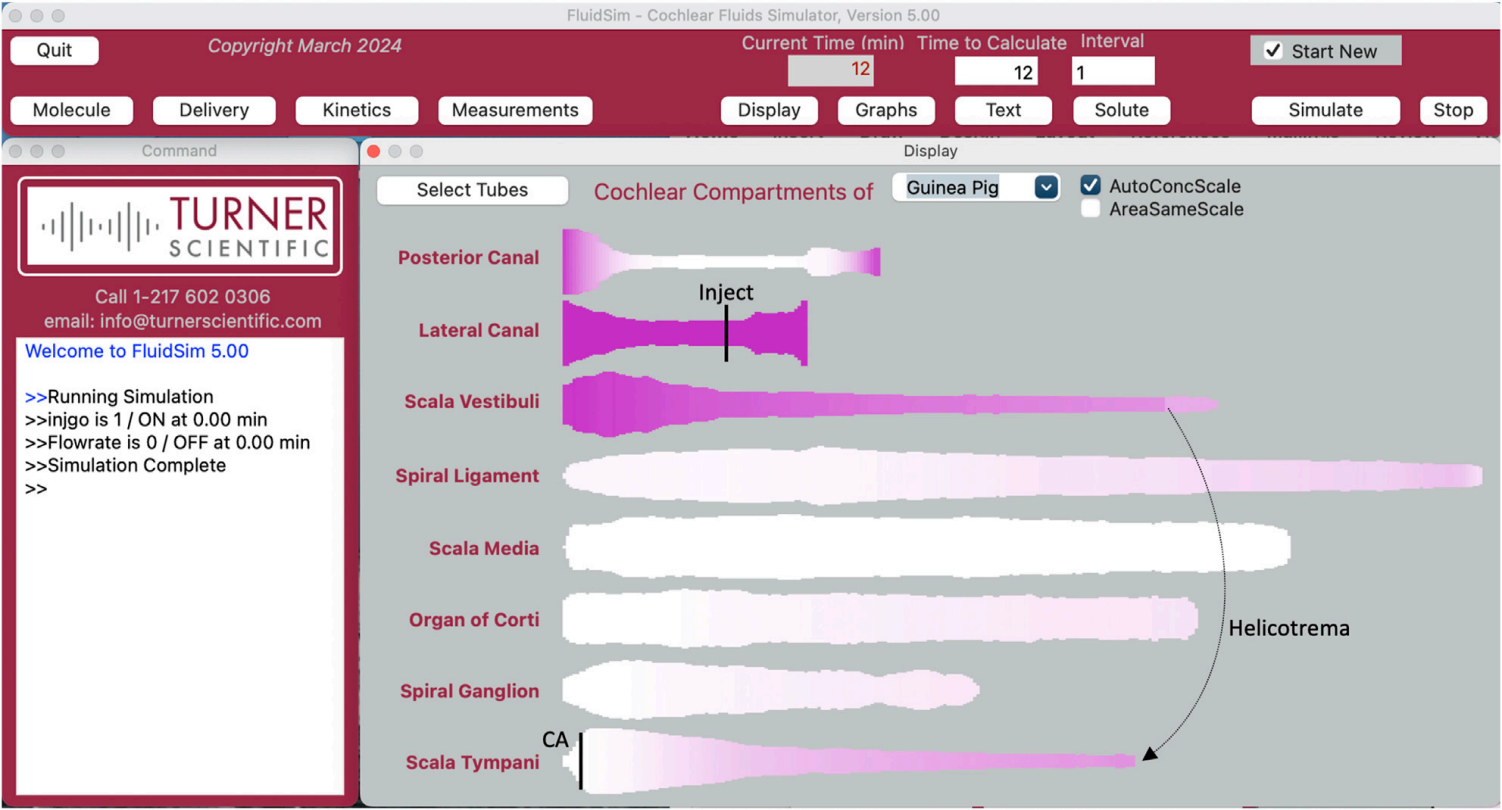
The FluidSim program as setup for lateral canal injection at 1 uL/min with the outlet at the cochlear aqueduct at the base of scala tympani (entry and exit sites indicated by black vertical lines). The flow path takes fluid each way along the lateral canal into the vestibule at the base of scala vestibuli, apically along scala vestibuli to the helicotrema and basally in scala tympani to the cochlear aqueduct (CA). Each compartment is displayed autoscaled to its largest area, distorting comparisons between compartments. Calculation has been stopped at 12 min. The lateral canal and scala vestibuli are loaded with drug and scala tympani is partially loaded, with a gradient from apex to base. Tissue compartments adjacent to the fluids are becoming loaded. The posterior canal (and anterior canal; not displayed), which are not in the injection flow path but become slowly loaded by diffusion. Injections of 60 min duration almost fully load the ear.

Experimental measurement of the rate of drug elimination from perilymph is technically challenging. Elimination cannot be quantified when drugs are applied to the middle ear. With local applications there are always gradients along the scalae, so it cannot be distinguished whether loss of drug from a region is directed to the vasculature or to a nearby region with lower concentration. In addition, middle ear applications rarely provide a stable entry rate as drug solutions in the middle ear decline rapidly with time. Instead, the method of choice to measure elimination from perilymph is to load the entire perilymphatic space with drug (Salt et al., 2012). This has been performed with identical methods for the 15 substances summarized here. In each case, drug solution was injected into the perilymph space of the lateral semicircular canal in guinea pigs. The injection pipettes were glass, with tip diameters of 30–50 μm, sealed into a lateral semi-circular canal (LSCC) fenestration with thin cyanoacrylate glue so that there was no fluid leakage. Injection was performed for 60 min at 1 μL/min. With the injection pipette sealed in place, the cochlear aqueduct provides the outlet for the injected volume. Drug solution passes in both directions around the LSCC to the vestibule, apically along SV, through the helicotrema and basally along ST to exit at the aqueduct. The flow path was confirmed by real-time measurements of marker concentration with ion-selective electrodes. (Salt et al., 2012). The 60 µL volume injected is approximately 4 times the perilymph volume of the guinea pig. For most drugs, this is sufficient to allow distribution into adjacent fluid and tissue spaces so that subsequent concentration declines represent loss from the ear (true elimination) rather than spread to regions of lower concentration. Nevertheless, when drugs are eliminated at high rates, even this injection rate and volume are not sufficient to fully load all the perilymphatic spaces. FluidSim replicates the drug loading protocol precisely, simulating the flow pattern along the scalae and taking into account the changing cross-sectional area with distance (Figure 2). Distribution in and out of adjacent tissue compartments is calculated based on the prevailing concentration gradient at each location with an exchange half time of 6 min for tissue compartments adjacent to ST and 110 min between the spiral ligament and SV. These rates are based on marker distribution measurements made in real time and for perilymph injection or sampling experiments with a variety of molecules (Salt et al., 1991; Salt et al., 2012; Salt et al., 2015). Elimination is determined using separate rates for SV and ST. The SV half-time is applied to SV, the vestibule and the perilymphatic spaces of each of the semi-circular canals. The ST half time is applied to ST and adjacent tissue spaces including the organ of Corti, the spiral ganglion and the auditory nerve.

After the drug injection, experiments included delay times of 0–4 h (a single delay time in each animal and delay times varied in different animals) to allow the influence of elimination to accumulate before perilymph sampling was performed. During this period FluidSim continues to calculate drug distribution between and along compartments according to prevailing concentration gradients, and to include the effects of CSF interactions (inflow and oscillatory exchange) in the basal turn of ST as quantified by Salt et al. (2015).

Experimentally, perilymph sampling was performed by removing the injection pipette from the LSCC, enlarging the fenestration slightly and collecting 20 × 1 µL samples sequentially with time, taking about 1–2 min per sample. The time taken to collect each sample was documented in an audio recording of the experiment. Collecting smaller samples improves the overall collection speed as the rate of fluid accumulation declines as the collection capillary fills. Perilymph is driven out of the fenestration by CSF entering at the aqueduct. For each sample, the precise volume was established by measuring the length of the sample immediately after collection using a calibrated reticule in a dissecting microscope. Samples were transferred to dilutent with a pair of samples added to each vial, providing 10 × 2 µL samples from each animal, diluted as required for analysis. This pairing of adjacent samples was performed to increase concentration, thereby improving resolution, and to minimize assay costs. FluidSim replicates the sampling process precisely, with the flow rate set by the sample volume divided by the time taken to collect it. Flow through the cochlea was simulated from the cochlear aqueduct, apically along ST, basally along SV and through the vestibule to the LSCC. At each array element along the flow pathway, drug exchange between the fluid space and adjacent tissue compartments was calculated (typically drugs diffusing out of the tissue spaces as concentration in the fluid spaces falls). Based on the flow rate, solute efflux at the collection site was summed, allowing the concentration of each of the 10 samples to be replicated based on the drug contents of the scalae.

The two perilymphatic regions not well loaded by the injection method are the anterior and posterior semi-circular canals, which are not subjected to the volume flows during drug injections or perilymph sampling. FluidSim includes the two canals in all calculations, including drug diffusion into the canal during and after drug delivery and diffusion out of the spaces during perilymph sampling.

For each drug, perilymph sample curves at each delay time were averaged across experiment replicates. Volumes and collection times for each of the 10 samples were also averaged. Averaged perilymph sample curves for each delay time were entered into an Excel spreadsheet. Separate runs of FluidSim were used to calculate the 10 samples for each of the delay times and transferred to the spreadsheet. Differences between calculated and measured concentrations for samples 1-4, calculated across all delay times, were used to adjust the SV elimination parameter. Differences between calculated and measured concentrations for samples 5-8 were used to adjust the ST elimination parameter. Calculations were repeated, adjusting elimination parameters incrementally until errors between calculated and measured samples were minimized. Parameters then represented the best fit to all delay times (equally weighted) included in the analysis. Examples of this fitting procedure have been shown in prior publications for dexamethasone (Salt et al., 2012, their Figure 8), for gentamicin (Salt et al., 2016; their Figure 4), triamcinolone-acetonide (Salt et al., 2019; their Figure 3A), and triamcinolone (Salt et al., 2019; their Figure 5A).

FluidSim simulations allowed PK properties to be derived by matching the simulated samples to the measured data. When appropriate elimination properties are defined, distribution characteristics for that drug can then be realistically quantified. In the drug injection experiment, gradients along the scalae can be calculated for any period without disruption by perilymph sampling. Distribution characteristics can then be more reliably predicted for other application protocols, such as local applications to the round window niche. The analyses in this study were performed with the most recent version of the FluidSim program (v 5.0) which is available for free download (Mac or PC) by following links from the Turner Scientific website (https://turnerscientific.com). All animal experiments analyzed here were performed under protocols approved by the Institutional Animal Care and Use Committees at Washington University or at Turner Scientific, as detailed in the appropriate publications.

## 3 Results

### 3.1 Influence of drug elimination on drug distribution in the human ear

**FIGURE 3:**
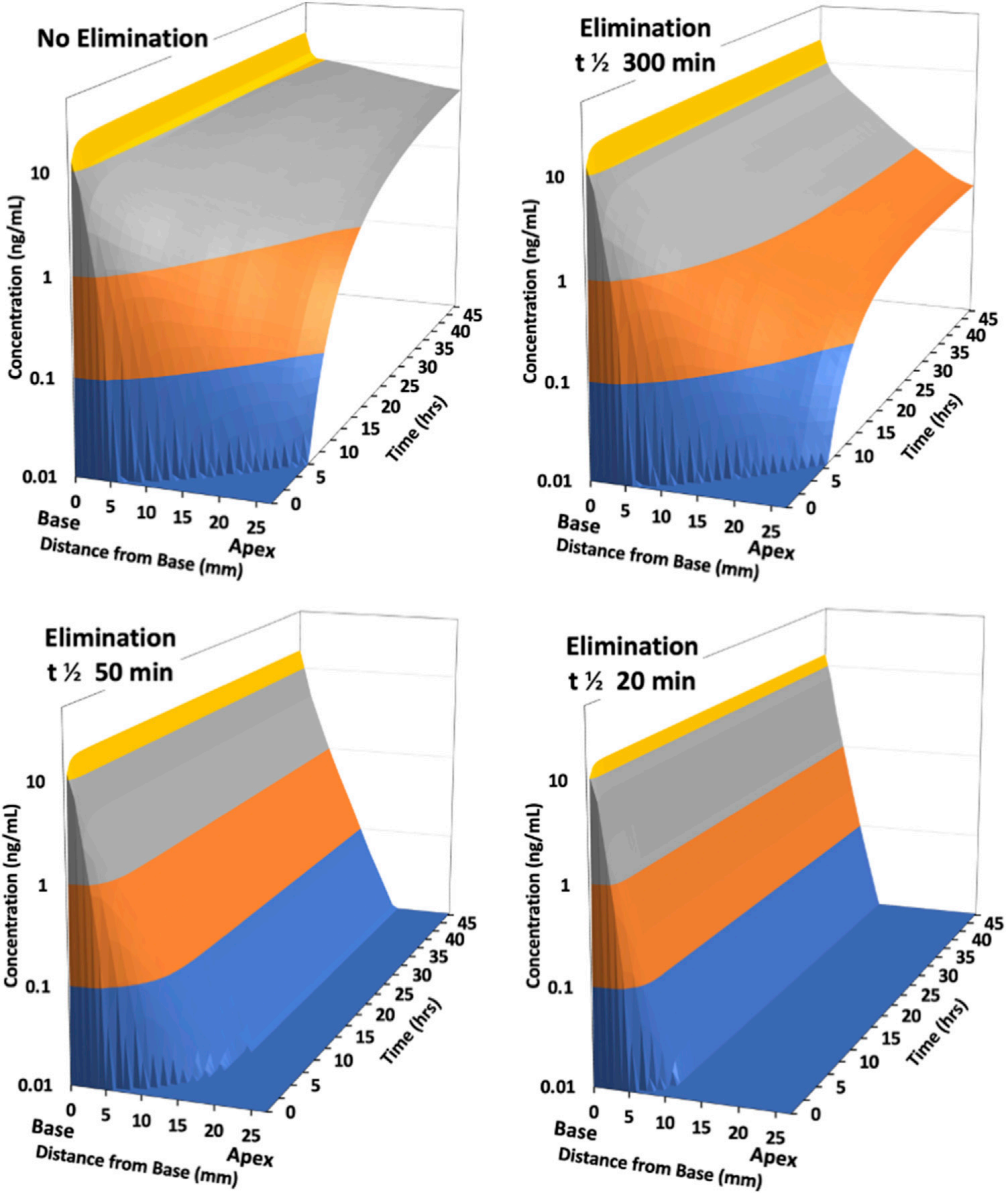
Calculated distribution of drug along scala tympani of the human ear with intratympanic administration as the half-time of elimination is varied. Distribution is calculated for a 48 h period with a sustained concentration in the middle ear, representing an idealized, best-case scenario. With no elimination from perilymph, drug reaches apical regions in 6–8 h. With a low rate of elimination (300 min half-time) results are initially similar, but a larger gradient along the scala persists with time. With elimination half-times of 50 min or 20 min, drug only reaches apical regions at very low concentration. A steady state is established with the drug gradient increasing with rapid elimination (i.e., with lower half-time).

The rate of elimination from perilymph plays a critical role in how the drug distributes along the fluid spaces of the ear. As the scalae of the human cochlea are substantially longer than those in most experimental animals, elimination is expected to generate a greater influence in the human. The effect of varying elimination for drug applied intratympanically to the human ear was calculated by FluidSim as illustrated in Figure 3.

As the half-time of elimination decreases (corresponding to faster elimination) the concentration of drug at the apical regions of the cochlea decreases. This is most apparent for longer times after application, where the gradient from base-to-apex becomes larger. With the most rapid elimination (half-times of 50 and 20 min) a steep gradient along the scala is rapidly established which does not decrease with exposure time. In apical regions only low concentrations can be achieved even for prolonged applications. This demonstrates that the ability of a drug to reach mid and apical cochlear regions at therapeutic concentration will depend highly on how rapidly the specific drug is eliminated from perilymph.

### 3.2 Measurement of drug elimination rate in Guinea pigs

**FIGURE 4:**
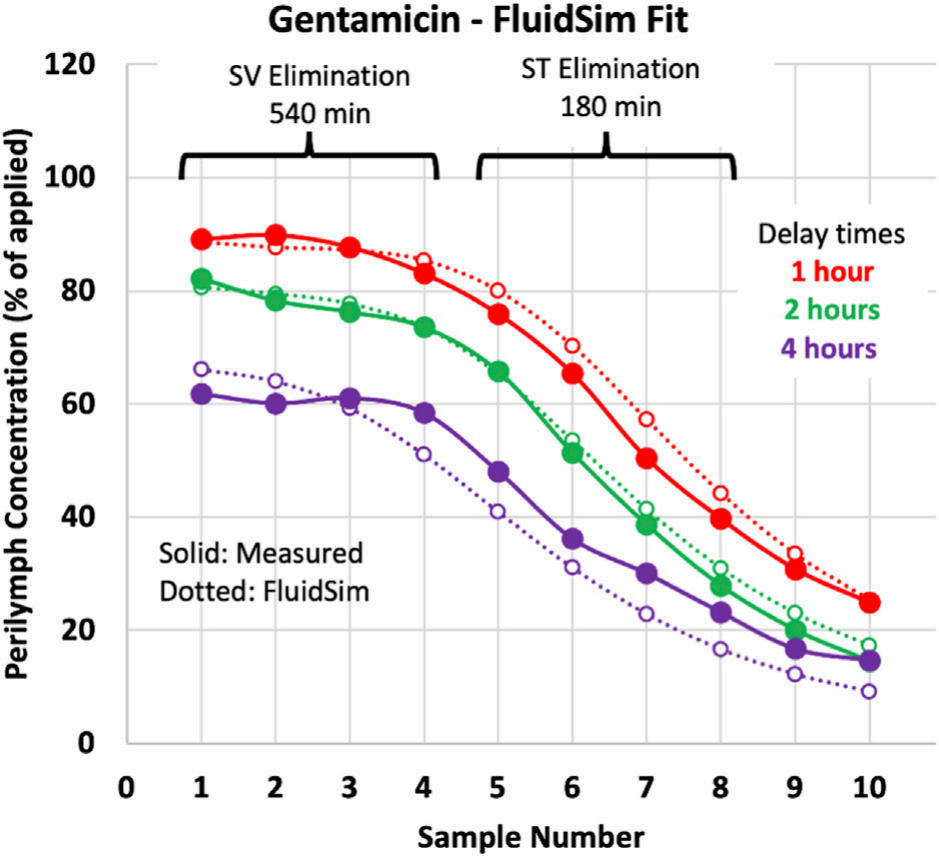
Perilymph elimination rate measurement in the guinea pig. The measured sample data (solid lines/symbols) are mean curves for gentamicin (from Salt et al., 2016). Dashed lines/open symbols show sample concentrations calculated by FluidSim simulations for the three delay times of the experiment. All three delay times were fitted simultaneously with a single set of parameters, so the curves are not the best fit to each individual condition.

Elimination rate was measured *in vivo* in guinea pigs by loading the perilymph and adjacent tissues with drug, followed by sequential perilymph sampling (10 × 2 µL samples from the LSCC) after various delay times. Figure 4 shows an example of the measurements and their analysis for gentamicin (from Salt et al., 2016).

As detailed in the methods, sample concentrations calculated by FluidSim were fitted to the measured samples by adjusting SV elimination to fit samples 1 to 4 and adjusting ST elimination to fit samples 5 to 8. The two elimination parameters were incrementally adjusted to fit all 3 delay conditions simultaneously, as shown by the dotted lines on the plot. Other relevant parameters in the FluidSim calculations, both fixed and varied, are summarized in Table 1.

**TABLE 1:** FluidSim parameters.

Inner ear dimensions data set	Guinea pig
Drug diffusion coefficient	Varied; Calculated from FW of drug where D = 7.45*(FW)^−0.38^
Scala vestibuli elimination half life	Varied to fit samples 1–4
Scala tympani elimination half life	Varied to fit samples 5–8
Perilymph-endolymph exchange half life	60 min for dexamethasone (based on prior measurements); None (unknown) for other drugs
Exchange half-time with tissue spaces adjacent to scala tympani	6 min
Scala vestibuli/spiral ligament exchange half-time	120 min
Perilymph-CSF exchange oscillation	3 nL/s
CSF flow into perilymph	30 nL/min

A similar FluidSim reanalysis of archival perilymph sample measurements for 13 drugs delivered with the same methodology in the guinea pig was performed. The elimination rates extracted by fitting FluidSim to the measured data are summarized in Table 2. Although the average elimination rate half-time was higher in SV compared to ST, the differences were not statistically significant (paired, t-test, *p* = 0.35), with 8 drugs showed a longer half-time in SV and 5 drugs showed a longer half-time in ST. We therefore combined ST and SV elimination rates as a log-scaled average, to represent the entire perilymph in a single value.

**TABLE 2:** Molecular properties of drugs and measured perilymph PK properties ranked in order of the combined rate of elimination from perilymph (longest halftime to shortest halftime). TPSA and WLOGP values were obtained from the SwissADME website (http://www.swissadme.ch/).

Drug	FW	TPSA	WLOGP	ST elim t½ (min)	SV elim t½ (min)	Perilymph elim t½ (min)	Data source
FITC Dextran	4,000	277.0	−7.70	170	10,000	1,304	2
Fluorescein (FITC)	332	81.7	4.43	490	890	660	1
Triamcinolone	394	115.1	1.04	1,250	280	592	4
FITC Dexamethasone	841	247.7	7.03	545	220	346	5
Gentamicin	478	199.7	−3.33	180	540	312	3
Texas Red Gentamicin	1,081	335.3	−0.63	250	154	196	5
Trimethylphenyl-Ammonium +	136	0.0	−1.11	183	71	114	1
Dexamethasone-phosphate	470	156.8	3.31	23	412	96	1
Dexamethasone	392	94.8	2.32	52	84	66	1
CHIR99021/FX03	465	115.2	4.30	90	28	50	6
Fluticasone propionate	501	106.0	5.69	19	48	30	7
Valproate/FX00	143	40.1	0.95	11	46	22	6
Triamcinolone-acetonide	435	93.1	2.84	9	33	17	4

Data sources for the reanalysis summarized in Table 2.

1) Salt AN, Hartsock JJ, Gill RM, Piu F, Plontke SK., Perilymph pharmacokinetics of markers and dexamethasone applied and sampled at the lateral semi-circular canal. J Assoc Res Otolaryngol. 2012 13(6): 771–783. PMCID: PMC3505589.

2) Salt AN, Gill RM, Hartsock JJ., Perilymph Kinetics of FITC-Dextran Reveals Homeostasis Dominated by the Cochlear Aqueduct and Cerebrospinal Fluid. J Assoc Res Otolaryngol. 2015; 16:357–371.

3) Salt AN, Hartsock JJ, Gill RM, King E, Kraus FB, Plontke SK., Perilymph pharmacokinetics of locally-applied gentamicin in the guinea pig. Hear Res. 2,016,342:101–111.

4) Salt AN, Hartsock JJ, Piu F, Hou J. Comparison of the pharmacokinetic properties of triamcinolone and dexamethasone for local therapy of the inner ear. Frontiers in Cellular Neuroscience 2019; 13, 347. https://www.frontiersin.org/article/10.3389/fncel.2019.00347.

5) Salt A, Hartsock J., Gill R., Zhang T., Li W., Dai C., Piu F., Hou J., Kraus F., Plontke S. Pharmacokinetic properties compared for native and fluorescent analogs of dexamethasone and gentamicin in the inner ear. 42nd Midwinter Research Meeting of the ARO, baltimore, February 2019, PS155.

6) McLean WJ, Herby J, Loose C, Hinton A, Lucchino D, Yang-Hood A, Schrader AD, Ohlemiller KK, Salt ANAUTHORID, Hartsock JJ, King S, Jackson L, Rosenbloom J, Aitee G, Bear M, Runge C, Gifford R, Lee D, Rauch S, Langer R, Karp J, LeBel C Improved Speech Intelligibility in Subjects With Stable Sensorineural Hearing Loss Following Intratympanic Dosing of FX-322, in a Phase 1b Study, Otol Neurotol. 2021 42(7):e849-e857.

7) Fluticasone study sponsored by Cochlear Limited, Sydney, Australia, 2018–2019; data used with permission.

In the table, drugs were ranked according to their perilymph elimination half-times with values ranging from 1,304 min for fluorescent-dextran to as low as 17 min for triamcinolone-acetonide. Notable elimination rates were 312 min for gentamicin, 66 min for dexamethasone and 17 min for triamcinolone acetonide. If the measured kinetics in guinea pigs are translatable to the situation when the same drugs are applied to humans, this would correspond to the 3D plots in Figure 3 approximating the distribution for gentamicin (300 min half time), dexamethasone (50 min half time) and triamcinolone-acetonide (20 min half time) respectively. Data were also analyzed for two molecules for which the results were proprietary and could not be included in the table for contractual reasons.

**FIGURE 5:**
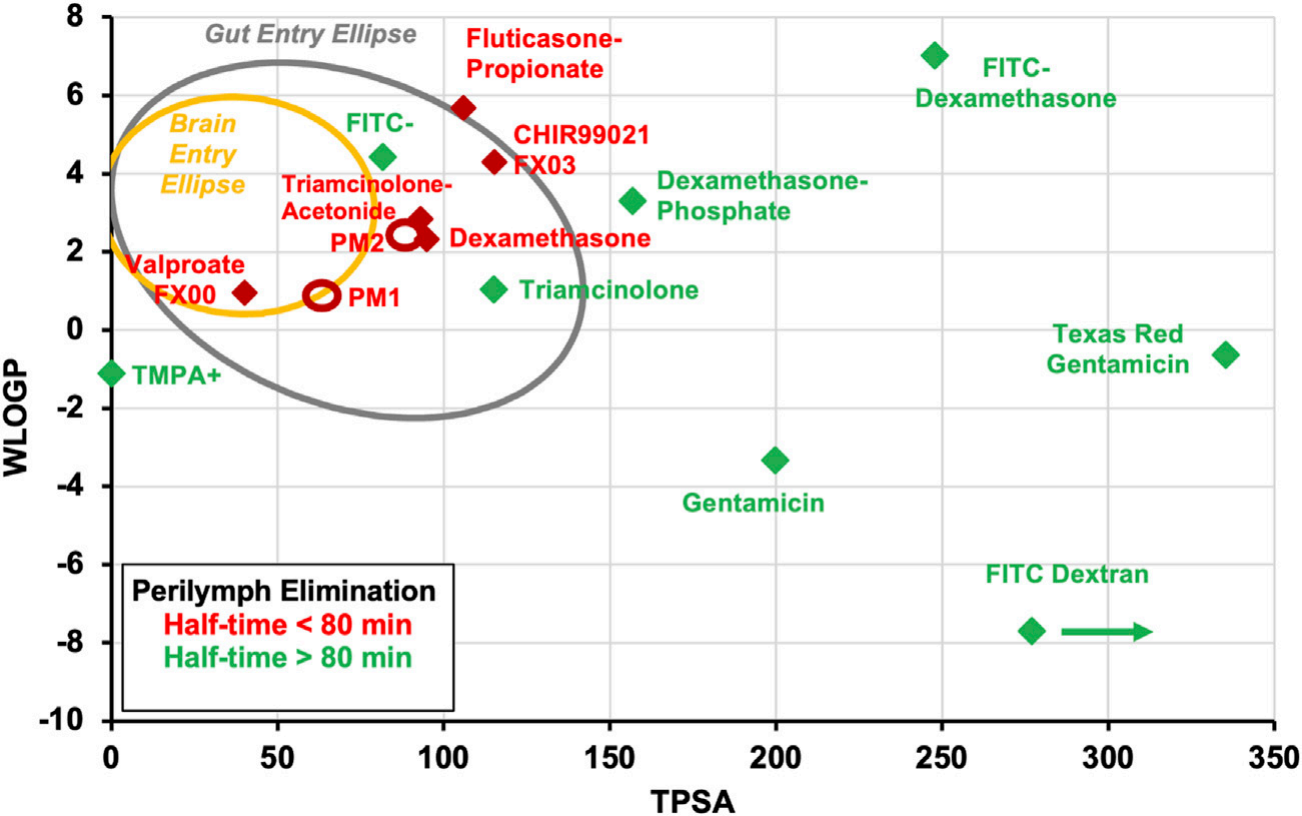
Measured perilymph kinetics summarized for the 15 drugs reanalyzed here. Data have been classified according to the measured perilymph elimination rate. Drugs with elimination half-times of less than 80 min are shown in red and those with elimination half-times higher than 80 min are shown in green. Most of the drugs that are eliminated rapidly are small and lipophilic (laying in the upper left quadrant of the plot). Drugs that are eliminated from perilymph more slowly tend to be larger, more polar (higher TPSA). An exception is the fluorescein anion (FITC). The TPSA for FITC-dextran lies off scale to the right as indicated by the arrow. The value plotted is for a 500 FW FITC-dextran because available software cannot calculate TPSA for the 4000 FW molecule. Ellipses PM1 and PM2 show the approximate molecular properties of two molecules for which elimination measurements are proprietary; both were eliminated with half-times well below 80 min.

The dependence of elimination rate on molecular properties is summarized for all 15 molecules in the egg plot shown in Figure 5. It is apparent that each of the 7 molecules exhibiting rapid elimination from perilymph were small and relatively lipophilic, located in the upper left quadrant of the graph. Drugs that were retained in perilymph were typically larger, more polar (higher TPSA), and hydrophilic. One prominent exception did exist, specifically fluorescein which is a small anion that was found not to be rapidly eliminated from perilymph (half-time 660 min). Fluorescein is actively transported across some epithelial boundaries which in this case maybe impeding efflux to the vasculature (Bresler et al., 1979).

## 4 Discussion

The primary conclusion from this analysis is that small, lipophilic molecules that are not retained in perilymph are unlikely to diffuse to apical cochlear regions of the human. They are therefore not well suited for treating cochlear disorders in humans with local applications. Intratympanic applications will treat the vestibular system and basal regions of cochlea, but drug may not reach apical regions of the cochlea at therapeutic concentration. In the human, the apical half of the cochlea codes many of the frequency regions important to speech (those below ∼2 kHz). Therefore, the inability of a drug to reach mid and apical regions can make it quite unsuitable for treating hearing disorders.

**FIGURE 6:**
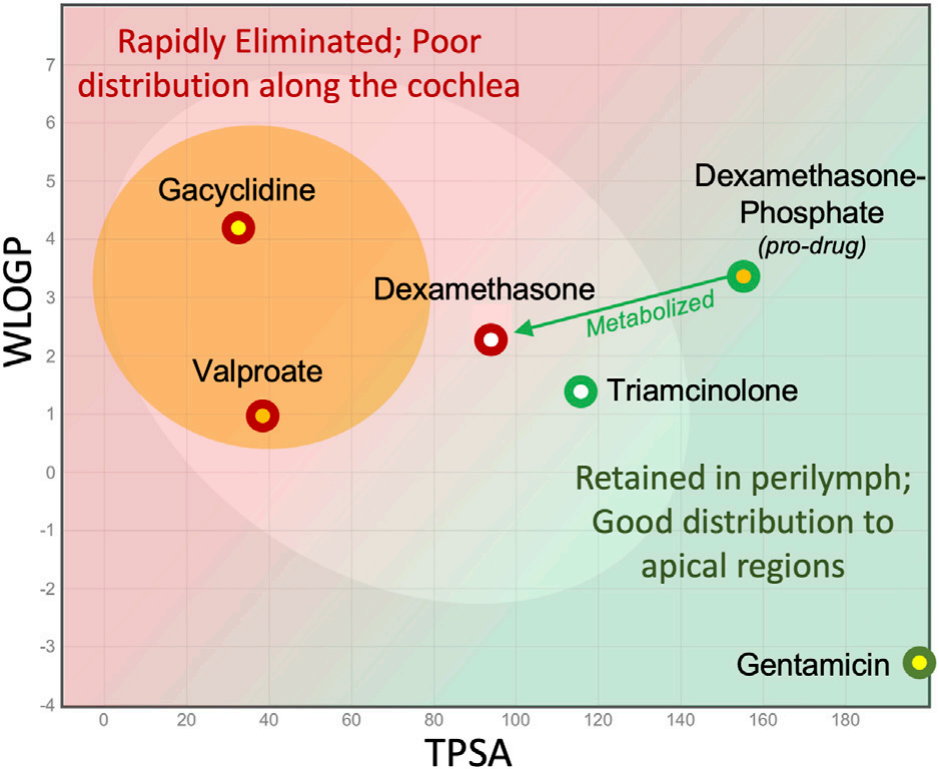
Consideration of molecular properties for local (intratympanic) therapy of the ear. Small lipophilic drugs may reach the entire ear when given systemically but with local applications are rapidly eliminated from perilymph, so they do not reach apical regions. Larger, more polar molecules are retained in perilymph for longer, allowing them to distribute more evenly along the length of the scalae.

All the locally applied drugs that have reached clinical trials in the past decade have been small lipophilic molecules. Outcomes in patients have been disappointing, to say the least, leading to a catastrophic loss of some of the companies involved. Otonomy had poor results with Otividex, a dexamethasone formulation used to treat Meniere’s patients, and with gacyclidine to treat tinnitus (both shown in Figure 6). Frequency Therapeutics had disappointing results with their FX322 formulation, of which valproate (shown on Figure 6) was a component. In their corporate presentations it was calculated that their FX-322 formulation would only treat frequency regions above 6 kHz in the human cochlea, leaving most of the frequencies important to hearing speech untreated. Frequency Therapeutics subsequently abandoned research in the hearing field. It should be appreciated that the relationships between elimination and molecular properties presented here were not available to the researchers at Otonomy or Frequency Therapeutics at the time the drugs were selected. Limited drug distribution is probably not the only factor underlying these failures. The chosen drugs may not have been delivered effectively, may not have been efficacious in humans or there may have been problems with patient selection for the clinical trials. Nevertheless, it remains to be demonstrated whether therapeutic concentrations of appropriate drugs were delivered effectively to mid and apical target regions of the human cochlea.

It remains a concern that many small, lipophilic molecules are being evaluated for potential therapy of the human cochlea. For example, there is considerable excitement about the efficacy of intratympanically-applied AC102 as a treatment to prevent hearing loss with stellar results in rats (Rommelspacher et al., 2024). The properties of AC102 place it in the middle of the “egg yolk” on the egg plot. Our analysis suggests it may be difficult for a drug with these properties to reach apical target tissues in the human when delivered locally. It is accepted that our prediction is based only on molecular properties and is not a good substitute for empirical measurements of elimination rate or of distribution properties of the drug. Our analysis suggests that supporting distribution studies of such molecules should be performed before proceeding too far along the pathway to local therapy in humans.

Whether any drug can reach apical target regions in humans with local applications is difficult to measure with current technology. Human temporal bone studies do not give a valid representation of drug distribution in the live human when elimination to the vasculature is present (Kang et al., 2016; Salt and Plontke, 2018). Perilymph cannot be sampled safely from apical cochlear regions of the living human. The only drug shown to reach apical regions of the human has been gentamicin. In one study gentamicin was delivered continuously over time with a round window catheter and pump, before the disastrous implications of this protocol were appreciated (Schoendorf et al., 2001). Eight of 11 patients went completely deaf (at all frequencies) on the treated side. This finding suggests that gentamicin had reached all frequency regions of the human ear at toxic concentration. The apical distribution of gentamicin in humans is consistent with the low rate of elimination measured in guinea pigs and on the molecular properties of the gentamicin molecule, shown in Figure 6.

In the absence of distribution studies in humans, drug gradients along the cochlea have been demonstrated in guinea pigs using sequential sampling from the cochlear apex to quantify the magnitude of gradients (Plontke et al., 2008). Sample measurements from these and other studies were used to refine FluidSim calculations. But the extrapolation from the guinea pig to the human carries a degree of uncertainty as the guinea pig cochlea is only about half the length of the human. There are significant anatomic differences between human and guinea pig cochleas that could influence the apical distribution of drugs. The human cochlea is wider and less tightly spiraled than the guinea pig, making ∼2.75 turns in humans compared to 3.5–3.75 turns in the guinea pig. It can be speculated that modiolar fluid pathways adjacent to the nerve may contribute more to apical drug distribution in humans. At present, however, there are no measurements to support such a speculation. Furthermore, the high vascularization of the auditory nerve and spiral ganglion probably already dominate drug elimination from perilymph. Fenestrations in the bone between ST and the spiral ganglion have been documented in both guinea pigs (Shepherd and Colreavy, 2004) and humans (Rask Andersen et al., 2006). It may therefore be expected that modiolar pathways would be subject to locally higher elimination rates than those measured in the perilymphatic scalae, limiting the contribution of the pathways to apical drug distribution.

A new approach to confirm drug distribution by measurements in human-sized ears has recently become available with the use of pigs or mini-pigs. The pig ear is approximately the same size as the human (Moatti et al., 2024) and perilymph can be collected from the cochlear apex by the sequential sampling technique, allowing gradients along ST of the pig to be directly quantified (Yildiz et al., 2023). It is expected that measurements in pigs and mini-pigs will be used to quantify distribution characteristics in a larger ear, allowing FluidSim predictions to be refined for larger ears. Especially when dealing with small, lipophilic molecules, it would seem both prudent and cost-effective to confirm that the drug is reaching the apical regions of the minipig cochlea before proceeding to clinical trials in humans.

Drugs that are well-suited for delivery to the brain (with properties located near or within the egg yolk) may well be appropriate for systemic delivery to the ear. In general, if the molecule is small/lipophilic enough to pass through the blood-brain barrier then it will also likely enter the ear with systemic application. Indeed, the blood-labyrinth barrier is anatomically less sophisticated than the blood-brain barrier (Salt and Hirose, 2018), lacking the astrocytes that are present in the blood-brain barrier. It can therefore be speculated that a broader range of molecules may enter the ear compared to the brain. However, there are currently no direct measurements to support this conjecture. Examples of small lipophilic therapeutic molecules that are delivered systemically include Ebselen/SPI 1005 (Kil et al., 2022) which is currently undergoing phase 3 clinical trials for the treatment of Meniere’s disease (Sound Pharmaceuticals, 2024), and Sens-401 (azasetrone) that reduces cisplatin-induced and noise-induced hearing loss in rats (Petremann et al., 2017; Petremann et al., 2019).

An equally important observation from this analysis is that larger, polar molecules (in the green area of Figure 6 and extending well beyond the right-hand axis) should be better suited for local delivery to the inner ear. It is important to appreciate that while there are certain molecules suitable for oral delivery and others suitable for systemic delivery to the brain, the range of molecules suitable for local delivery to the ear will be completely different and occupy a completely different area of the egg plot. This includes thousands of molecules that fail Lipinsky’s rules, have FWs >500, and are categorized as “not druglike” (not suitable for oral delivery). Many such drugs have been dismissed for further development and largely ignored by the pharmaceutic industry. This presents an enormous opportunity to screen drug libraries for large molecules with appropriate physical and pharmaceutic properties for the therapy of hearing disorders. Suitable polar molecules cover an enormous range, including biologics and antibodies.

An important related consideration is whether larger, polar molecules will enter perilymph by passing through the round window membrane. Even though polar, gentamicin, dextran and other polar molecules pass through the round window membrane. If entry becomes insufficient, then excipients may be used to transiently permeabilize the membrane allowing more drug to enter (Li et al., 2018). The technical aspects of direct injections into perilymph are also being developed, primarily to enable gene therapy (Regeneron press release, 2023; Lv et al., 2024). There are also major efforts to develop intracochlear drug delivery devices which may overcome some of the distribution limitations by releasing drug more apically within the cochlea (Dhanasingh and Hochmair, 2021; Lehner et al., 2022; Prenzler et al., 2023).

Dexamethasone deserves special consideration as it lies in the “gray zone” of Figure 6. It has been shown that the similar steroid, triamcinolone, has lower elimination rates and will therefore distribute apically more effectively (Salt et al., 2019). However, clinical studies show that dexamethasone is considerably more potent than triamcinolone base (to be distinguished from triamcinolone-acetonide), therefore requiring a higher concentration in apical cochlear regions to be equivalent. Although dexamethasone has less than ideal PK properties, it probably remains the best available corticosteroid for local inner ear therapy.

The search for more appropriate molecular properties also opens the possibility for molecular engineering, modifying existing molecules to improve their PK properties. Fluorescent dexamethasone binds to the glucocorticoid receptor and is shown here (in Table 2) to have substantially better PK properties than native dexamethasone. In contrast dexamethasone-phosphate (the most common form of dexamethasone used clinically) is a pro-drug and does not bind to the glucocorticoid receptor. Rather (as shown in Figure 5) it is rapidly metabolized to dexamethasone. Fluorescent dexamethasone may not be an appropriate candidate for inner ear therapy but serves to demonstrate that modification of drug molecules to improve the PK characteristics in the cochlea may have considerable potential.

## Data Availability

The raw data supporting the conclusions of this article will be made available by the authors, without undue reservation.

## Abbreviations

LSCC, lateral semi-circular canal; PK, pharmacokinetic; ST, scala tympani; SV, scala vestibuli; TPSA, topological polar surface area.
