# Supplementary material for: Drug selection for inner ear therapy
Source: Front Pharmacol. 2024 Oct 17;15:1452927. doi: 10.3389/fphar.2024.1452927 (PMC11525065; doi:10.3389/fphar.2024.1452927)
Supplement: Supplementary file 1 [file Table1.docx]

Supplementary Material

Table S1: Published studies in which FluidSim output was compared to measured data, allowing parameter extraction and optimization of simulator algorithms.

*Columns show the drug, species, and specific distribution processes under focus in each of the studies.*

| **Molecule** | **Species** | **Diffusion + flow** | **Elimin-ation** | **Inter-compart-ment spread** | **Peri injection / Elution** | **Entry from Middle Ear** | **Reference** |
| --- | --- | --- | --- | --- | --- | --- | --- |
| TMA | Guinea Pig | X |  |  | X |  | Salt et al., 1986 |
| TMA | Guinea Pig | X |  |  | X |  | Salt & Thalmann, 1986 |
| TMPA | Guinea Pig | X | X |  | X |  | Ohyama et al., 1988 |
| TEA | Guinea Pig | X |  |  | X |  | Salt & Thalmann, 1988 |
| TMA, TEA, TMPA | Guinea Pig | X | X |  | X |  | Salt & Thalmann, 1989 |
| TMPA | Guinea Pig |  | X | X | X |  | Salt et al., 1991a |
| TMPA | Guinea Pig | X |  |  | X |  | Salt et al., 1991b |
| TMA | Guinea Pig | X | X |  | X |  | Salt & DeMott 1992 |
| AsF6 | Guinea Pig | X | X |  | X |  | Salt & DeMott, 1994 |
| AsF6 | Guinea Pig | X | X |  | X |  | Salt et al., 1995 |
| TMPA | Guinea Pig | X | X |  | X |  | Salt & DeMott, 1997 |
| TMPA | Guinea Pig | X | X | X |  | X | Salt & Ma, 2001 |
| Gent | Guinea Pig | X | X | X |  | X | Plontke et al., 2002 |
| MPred, Pred | Guinea Pig |  | X | X |  | X | Plontke et al., 2003 |
| TMPA | Guinea Pig | X | X | X |  | X | Salt et al., 2003 |
| Dex, Fluor | Guinea Pig |  | X |  |  | X | Hahn et al., 2006 |
| TMPA | Guinea Pig |  | X | X |  | X | Mynatt et al., 2006 |
| TMPA | Guinea Pig | X | X | X | X | X | Salt et al., 2006 |
| Gent | Guinea Pig | X | X |  |  | X | Plontke et al., 2007 |
| TMPA | Guinea Pig | X | X | X | X |  | Salt et al., 2007 |
| TMPA | Guinea Pig |  |  |  |  | X | Mikulec et al., 2008 |
| Dex | Guinea Pig |  | X |  |  | X | Plontke et al., 2008a |
| Mpred | Human |  | X |  |  | X | Plontke et al., 2008b |
| Gent, TMPA | Guinea Pig |  | X |  |  | X | Mikulec et al., 2009 |
| Gadolinium | Guinea Pig |  |  |  |  | X | King et al., 2011 |
| Dex | Guinea Pig |  | X | X |  | X | Salt et al, 2011 |
| TMPA | Guinea Pig | X | X | X | X | X | Salt et al., 2012a |
| Dex, Dex-P, Fluor, TMPA | Guinea Pig | X | X |  | X |  | Salt et al., 2012b |
| Dex | Guinea Pig | X | X | X | X |  | Hahn et al., 2012 |
| Gent | Guinea Pig |  |  | X |  |  | Hahn et al., 2013 |
| TMPA | Guinea Pig | X |  |  | X | X | King et al., 2013 |
| Fluor | Mouse |  |  |  |  |  | Hirose et al., 2014 |
| FITC-Dextran | Guinea Pig | X | X | X | X |  | Salt et al., 2015 |
| Gent | Guinea Pig | X | X |  | X | X | Salt et al., 2016 |
| FITC-Dextran | Guinea Pig | X | X |  | X |  | Salt et al., 2017 |
| Dex, Dex-P | Guinea pig, Human |  |  |  |  | X | Salt et al. 2018 |
| TriamX, TriamA | Guinea Pig |  | X |  | X | X | Salt et al., 2019 |
| CHIR99021, Valproate | Guinea Pig, Human |  | X |  |  | X | McLean et al., 2021 |
| FITC-Dextran | Macaque |  |  | X | X |  | Manrique-Huarte et al., 2021 |
| Dex, Dex-P | Domestic Pig |  |  |  |  | X | Moatti et al., 2024 |

**Abbreviations used in the table:** Dex: Dexamethasone; Dex-P: Dexamethasone-phosphate; Fluor: Fluorescein; FITC-Dextran: Fluorescent labeled dextran, FW 4000; Gent: Gentamicin; Mpred: Methylprednisolone; Pred: Prednisolone; TMA: Tetramethylammonium; TMPA:Trimethylphenylammonium; TEA: Tetraethylammonium; AsF6:Arsenic hexafluoride; TriamX: Triamcinolone; TriamA: Triamcinolone Acetonide

**References**

Salt, A.N., Thalmann, R., Marcus, D.C. and Bohne, B.A.: Direct measurement of longitudinal endolymph flow rate in the guinea pig cochlea. Hearing Res. 23:141‑151, 1986.

Salt, A.N. and Thalmann, R.: New concepts regarding the volume flow of endolymph and perilymph. Adv. Oto‑Rhino‑Laryngol. 37:11‑17, 1987.

Ohyama, K., Salt, A.N. and Thalmann, R.: Volume flow rate of perilymph in the guinea‑pig cochlea. Hearing Res. 35:119‑130, 1988.

Salt, A.N. and Thalmann, R.: Interpretation of endolymph flow results. Hearing Res. 33:279 281, 1988.

Salt, A.N. and Thalmann, R.: Rate of longitudinal flow of cochlear endolymph. In: Ménière's Disease. Ed., J.B. Nadol, Kugler, Amsterdam, pp. 69‑73, 1989.

Salt, A.N., Ohyama, K and Thalmann, R.: Radial communication between the perilymphatic scalae of the cochlea. I. Estimation by tracer perfusion. Hearing Res. 56:29‑36, 1991a

Salt, A.N., Inamura, N., Thalmann, R. and Vora, A.R.: Evaluation of procedures to reduce fluid flow in the fistulized guinea pig cochlea. Acta Otolaryngol. 111:899 907, 1991b

Salt, A.N. and DeMott, J.: Tetramethylammonium for in vivo marking of the cross sectional area of the scala media in the guinea pig cochlea. Archives Oto Rhino Laryngology 249:157 163, 1992.

Salt, A.N. and DeMott, J.D.: Time course of endolymph volume increase in experimental hydrops measured in vivo with an ionic volume marker. Hearing Res. 74:165-172, 1994.

Salt, A.N., DeMott, J.E. and Kimura, R.S.: Comparison of endolymph cross-sectional area measured histologically with that measured in vivo with an ionic volume marker. Annals of Otol-Rhinol-Laryngol. 104:886-894, 1995

Salt, A.N. and DeMott, J.E. Longitudinal endolymph flow associated with acute volume increase in the cochlea. Hearing Research 107, 29-40, 1997

Salt, A.N. and Ma, Y. Quantification of solute entry into cochlear perilymph through the round window membrane. Hear. Res. 154, 88-97, 2001.

Plontke, S.K.R., Wood, A.W., Salt, A.N. Analysis of gentamicin kinetics in fluids of the inner ear with round window administration. Otology and Neurotology 23: 967-974 2002.

Plontke, S.K.R. and Salt, A.N. Quantitative interpretation of corticosteriod pharmacokinetics in inner ear fluids using computer simulations. Hear Res 182, 34-42, 2003.

Salt, A.N., Kellner, C., Hale, S. Contamination of perilymph sampled from the basal cochlear turn with cerebrospinal fluid. Hear. Res. 182, 24-33, 2003.

Hahn, H., Kemmerer, B., DiMauro, A., Salt, A.N., Plontke, S. Cochlear microdialysis for quantification of dexamethasone and fluorescein entry into scala tympani during round window administration. Hearing Research 212:236-244, 2006.

Mynatt, R., Hale, S.A., Gill, R.M., Plontke, S.K.R., Salt, A.N. Demonstration of a longitudinal concentration gradient along scala tympani by sequential sampling of perilymph from the cochlear apex. J Assoc Res Otolaryngol. 7:182-193, 2006.

Salt, A.N., Hale, S.A., Plontke, S.K.R. Perilymph sampling from the cochlear apex: A reliable method to obtain higher purity perilymph samples from scala tympani. Journal of Neuroscience Methods 153: 121-129, 2006

Plontke SK, Mynatt R, Gill RM., Salt AN. Concentration gradient along scala tympani following the local application of gentamicin to the round window membrane. Laryngoscope. 117:1191-1198, 2007.

Salt, A.N., Sirjani, D.B., Hartsock, J.J., Gill, R.M., Plontke, S.K. Marker Retention in the Cochlea Following Injections through the Round Window Membrane. Hear Res. 2007;232:78-86.

Mikulec AA, Hartsock JJ, Salt AN. Permeability of the round window membrane is influenced by the composition of applied drug solutions and by common surgical procedures, Otol Neurotol 29:1020-1026, 2008.

Plontke, S.K., Biegner, T., Kammerer, B., Delabar, U., Salt, A.N. Dexamethasone concentration gradients along scala tympani after application to the round window membrane. Otology & Neurotology 29:401-406, 2008a.

Plontke SK, Mikulec AA, Salt AN. Rapid clearance of methylprednisolone after intratympanic application in humans. (Letter to the Editor) Otology & Neurotology Otol Neurotol. 2008b 29:732-733;

Mikulec AA, Plontke SK, Hartsock JJ, Salt AN Entry of substances into perilymph through the bone of the otic capsule following intratympanic applications in guinea pigs: Implications for local drug delivery in humans. Otol Neurotol. 30:131-138, 2009.

King EB, Salt AN, Eastwood HT, O'Leary SJ. Direct entry of gadolinium into the vestibule following intratympanic applications in Guinea pigs and the influence of cochlear implantation. J Assoc Res Otolaryngol. 2011 Dec;12(6):741-51.

Salt AN, Hartsock JJ, Plontke SK, LeBel C, Piu F. Distribution of dexamethasone and preservation of inner ear function following intratympanic delivery of a gel-based formulation. Audiology & Neurotology 2011; 16:323-335.

Salt AN, King EB, Hartsock JJ, Gill RM, O'Leary SJ. Marker entry into vestibular perilymph via the stapes following applications to the round window niche of guinea pigs. Hear Res. 2012a 283:14-23.

Salt AN, Hartsock JJ, Gill RM, Piu F, Plontke SK. Perilymph pharmacokinetics of markers and dexamethasone applied and sampled at the lateral semi-circular canal. J Assoc Res Otolaryngol. 2012b 13(6): 771–783.

Hahn H, Salt AN, Beigner, T, Kammerer B, Delabar U, Hartsock J, Plontke SK. Dexamethasone levels and base to apex concentration gradients in scala tympani perilymph following intracochlear delivery in the guinea pig Otol Neurotol. 33:660-665, 2012.

Hahn H, Salt AN, Schumacher U, Plontke SK Gentamicin concentration gradients in scala tympani perilymph following systemic applications Audiol Neurootol. 2013;18(6):383-91.

King EB, O’Leary SJ, Hartsock JJ, Salt AN. Influence of Cochleostomy and Cochlear Implant Insertion on Drug Gradients following Intratympanic Application in Guinea Pigs. Audiol Neurootol. 2013; 18:307-316.

Hirose K, Hartsock JJ, Johnson S, Santi P, Salt AN. Systemic lipopolysaccharide compromises the blood-labyrinth barrier and increases entry of serum fluorescein into the perilymph. J Assoc Res Otolaryngol. 2014 15:707-719.

Salt AN, Gill RM, Hartsock JJ. Perilymph Kinetics of FITC-Dextran Reveals Homeostasis Dominated by the Cochlear Aqueduct and Cerebrospinal Fluid. J Assoc Res Otolaryngol. 2015;16:357-371.

Salt AN, Hartsock JJ, Gill RM, King E, Kraus FB, Plontke SK. Perilymph pharmacokinetics of locally-applied gentamicin in the guinea pig. Hear Res. 2016 342:101-111.

Salt A, Hartsock J, Gill R, Smyth D, Kirk J, Verhoeven K. Perilymph pharmacokinetics of marker applied through a cochlear implant in guinea pigs. PLoS One. 2017 12(8):e0183374.

Salt AN, Hartsock JJ, Piu F, Hou, J. Dexamethasone and dexamethasone-phosphate entry into perilymph compared for middle ear applications in guinea pigs. Audiol Neurootol. 2018;23:245-257.

Salt AN, Hartsock JJ, Piu F, Hou J. Comparison of the pharmacokinetic properties of triamcinolone and dexamethasone for local therapy of the inner ear. Frontiers in Cellular Neuroscience 2019; 13, 347. https://www.frontiersin.org/article/10.3389/fncel.2019.00347

McLean WJ, Herby J, Loose C, Hinton A, Lucchino D, Yang-Hood A, Schrader AD, Ohlemiller KK, Salt AN, Hartsock JJ, King S, Jackson L, Rosenbloom J, Aitee G, Bear M, Runge C, Gifford R, Lee D, Rauch S, Langer R, Karp J, LeBel C Improved Speech Intelligibility in Subjects With Stable Sensorineural Hearing Loss Following Intratympanic Dosing of FX-322 in a Phase 1b Study, Otology & Neurotology: February 22, 2021 - doi: 10.1097/MAO.0000000000003120.

Manrique-Huarte R, Linera-Alperi MA, Parilli D, Rodriguez JA, Borro D, Dueck WF, Smyth D, Salt A, Manrique M. Inner ear drug delivery through a cochlear implant: Pharmacokinetics in a Macaque experimental model. Hear Res. 2021 404:108228. doi: 10.1016/j.heares.2021.108228. Epub 2021 Mar 19. PMID: 33784550.

Moatti A, Connard S, De Britto N, Dunn WA, Rastogi S, Rai M, Schnabel LV, Ligler FS, Hutson KA, Fitzpatrick DC, Salt A, Zdanski CJ, Greenbaum A. Surgical procedure of intratympanic injection and inner ear pharmacokinetics simulation in domestic pigs. Front Pharmacol. 2024 Jan 26;15:1348172. doi: 10.3389/fphar.2024.1348172.
